# Anti‐MDA5 Dermatomyositis Presenting With Lupus‐Like Mucocutaneous Features and Inflammatory Arthritis: A Case Report

**DOI:** 10.1155/crii/7236906

**Published:** 2026-09-26

**Authors:** Laras Budiyani, Dinisa Diah Winari, Agnes Theodora, Suzy Maria, Sukamto Koesnoe, Anshari Saifuddin Hasibuan, Evy Yunihastuti, Findy Prasetyawaty, Luh Ari Indrawati, Eyleny Meisyah Fitri

**Affiliations:** ^1^ Department of Internal Medicine, Faculty of Medicine, Universitas Indonesia, Cipto Mangunkusumo National General Hospital, Jakarta, Indonesia, ui.ac.id; ^2^ Department of Internal Medicine, Cipto Mangunkusumo National General Hospital, Jakarta, Indonesia; ^3^ Department of Neurology, Cipto Mangunkusumo National General Hospital, Jakarta, Indonesia; ^4^ Department of Dermatology and Venereology, Cipto Mangunkusumo National General Hospital, Jakarta, Indonesia

**Keywords:** case report, dermatomyositis, disease mimicry, MDA-5, overclassification, rheumatoid arthritis, SLE

## Abstract

Understanding diagnosis and management of specific autoimmune diseases is based on existing classifications or diagnostic criteria. However, patients frequently present with broad, multisystem features that mimic multiple autoimmune diseases. Recognizing clinical mimicry is crucial because overclassifying these features as distinct coexisting diseases often presents a diagnostic challenge. We report the case of a 36‐year‐old man who presented with progressive weakness. The patient initially complained of facial rash and skin lesions, which then developed into tetraparesis, arthralgia, and difficulty swallowing. Physical examination revealed various skin and musculoskeletal manifestations, including erythematous plaques on the face without involving the nasolabial folds, discoid rash, heliotrope rash, Gottron’s sign, and multiple ulcers. Neurological examination showed decreased muscle strength and motor lesions. Laboratory results showed an increase in inflammatory markers, positive antinuclear antibodies (ANAs) as well as positive myositis‐specific antibodies for melanoma differentiation‐associated protein (MDA)‐5. Electromyography (EMG) examination showed findings consistent with myogenic lesions. Chest CT scan showed interstitial lung disease (ILD), and hand radiography showed juxta‐articular wrist osteopenia consistent with early signs of inflammatory arthritis. Based on these findings, the patient was diagnosed with MDA‐5 dermatomyositis (DM) with lupus‐like features and inflammatory arthritis, representing a high‐risk DM phenotype that clinically mimics a triple overlap syndrome, rather than three distinct confirmed diseases. Initial treatment involved the administration of high‐dose methylprednisolone followed by a gradual dose reduction, hydroxychloroquine, and mycophenolate sodium after the secondary skin infection resolved. The patient received multidisciplinary care involving various specialties. After treatment, the patient’s motor weakness improved. Distinguishing severe phenotypes of connective tissue diseases (CTDs) from true overlapping syndromes is a diagnostic challenge due to overlapping symptoms and laboratory findings. Accurate diagnosis requires a comprehensive clinical and serological evaluation, as well as a deep understanding of the classification criteria for each disease to avoid overclassification. Early diagnosis and aggressive treatment targeting the primary driving pathology can improve the prognosis and quality of life of patients.

## 1. Introduction

Connective tissue diseases (CTDs) are a group of autoimmune diseases that can impact various organs and manifest with a range of symptoms. Common CTDs include conditions like rheumatoid arthritis (RA), systemic lupus erythematosus (SLE), Sjögren’s syndrome, systemic sclerosis, and dermatomyositis (DM). Each CTD has specific diagnostic criteria based on clinical and laboratory findings. While some individuals may present with characteristics of multiple CTDs—a phenomenon referred to as “Overlap Syndrome”—severe phenotypes of a single primary disease can frequently mimic multiple distinct conditions. Diagnosing coexisting autoimmune diseases poses a challenge, and the failure to recognize clinical mimicry often leads to diagnostic overclassification and misguided treatment approaches [1].

## 2. Case Presentation

A 36‐year‐old Indonesian male patient presented with worsening muscle weakness, with standing and walking difficulty for a week, accompanied by difficulty in swallowing. There was swelling and pain in the joints that had worsened 3 weeks prior. Since 2023, the patient had complained of a facial rash accompanied by sores on the back, elbows, and back of the hands and was initially suspected of having seborrheic dermatitis. Since 2024, the patient had frequently complained of pain in the hands and feet accompanied by stiffness and cramps in the morning, and there was weakness when lifting objects.

**Figure 1:**
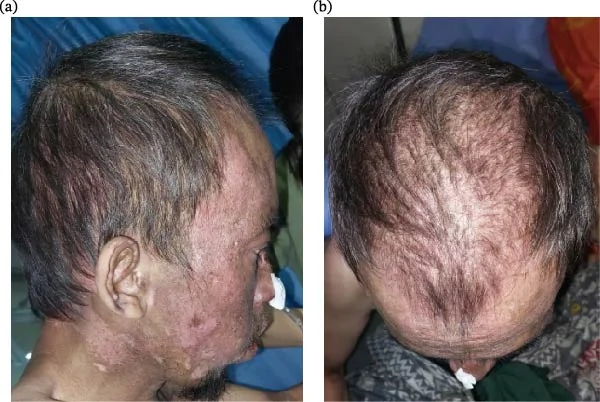
(a) Annular erythematous patch of varying size followed by follicular hyperkeratosis, hyperpigmentation at the periphery, with central hypopigmentation seen in the face and scalp. (b) Non scarring alopecia.

**Figure 2:**
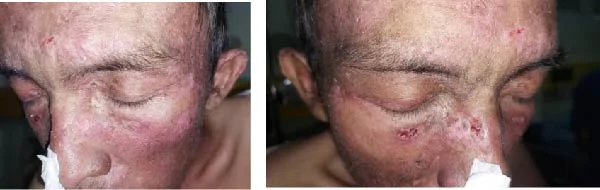
Heliotrope sign (violet to erythematous rash affecting the upper eyelids) and multiple erythematous plaques of irregular size on the forehead, eyebrows, and malar area with nasolabial sparing.

**Figure 3:**
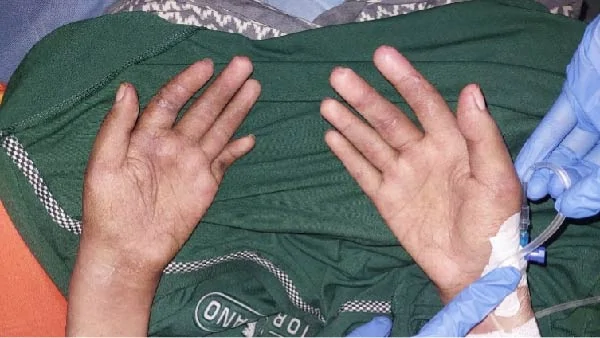
Inverse Gottron’s sign. Erythematous papules and macules seen in the palmar aspect of the finger creases.

Physical examination revealed multiple erythematous plaques of irregular size on the forehead, eyebrows, and malar area with nasolabial sparing, scalp (Figure 1a and Figure 2), anterior neck to upper chest, back, nonscarring alopecia (Figure 1b). In addition, heliotrope sign (Figure 2) and inverse Gottron’s papules (painful erythematous papules and macules involving the palmar aspect of the finger creases) (Figure 3). There were erythematous lesions with follicular hyperkeratosis and peripheral hyperpigmentation in the jaw area to the auricles, mimicking discoid lupus (Figure 1a).

**Figure 4:**
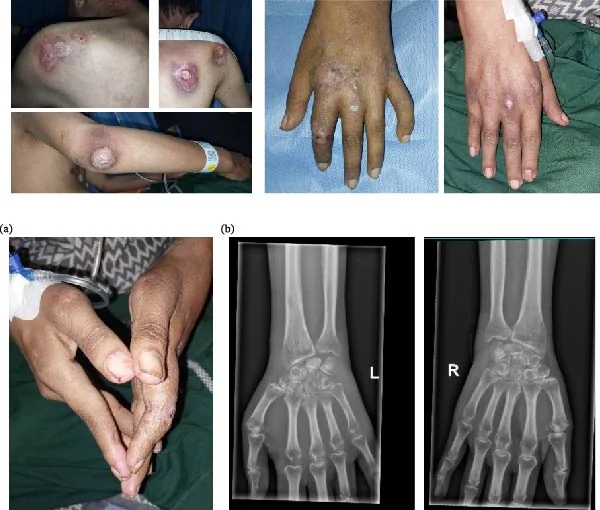
Multiple ulcers shown on right scapula, left and right shoulders and elbow and metacarpophalangeal joints of the fingers. (a) Hand deformity showing flexion at the PIP joint and hyperextension at the DIP joint of the 2^nd^ left hand digit. (b) Radiographic image showing juxta‐articular wrist osteopenia.

Multiple ulcers were seen in the right scapular region and left shoulder as well as the right elbow (Figure 4). In the neck, there was decreased motor function in the flexors compared to that in the extensors. In the upper extremities, there was decreased motor strength in flexion and extension of the wrists and digits (not in the elbows and shoulders), while in the lower extremities, there was decreased motor strength in hip adduction‐abduction, knee flexion, and extension, but not in the distal part. Tenderness was found in bilateral elbows, bilateral wrists, as well as swelling in metacarphophalangeal (MCP) joint of the 2^nd^, 3^rd^, and 4^th^ left digits, MCP joint of the 2^nd^–5^th^ right hand digits, proximal interphalangeal (PIP) of the 2^nd^, 3^rd^, and 5^th^ left hand digits and 2^nd^–4^th^ right hand digits and flexion at the PIP joint and hyperextension at the distal interphalangeal joint (DIP) of the 2^nd^ left hand digit (Figure 4a).

**Figure 5:**
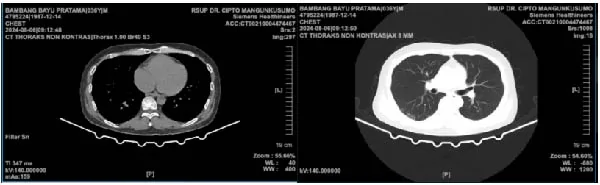
Chest CT scan results showed reticular opacities and surrounding ground‐glass opacities in both lungs.

Based on the medical history and electromyography (EMG) examination, axonal lesions of the motor nerve fibers were found, consistent with myogenic lesions. Additional diagnostic investigations, such as muscle MRI, muscle biopsy, or extended muscle enzyme testing, were not performed, limiting full confidence in the scope of the diagnostic workup. Laboratory examinations were performed, and the results showed normal complete blood count, normal CK (which is an important and recognized feature of melanoma differentiation‐associated protein (MDA)‐5‐associated DM despite significant weakness), normal electrolytes, elevated ESR (54 mm, normal range 0–15) and CRP (7.3 mg/L, normal range <5 mg/L), elevated transaminases, decreased C3 (48 mg/dL normal range 90–180 mg/dL), elevated rheumatoid factor (RF; 153.1 IU/mL, normal range <15.9 IU/mL), negative skin BTA for Hansen’s disease, positive antinuclear antibody (ANA) 1/320 with a coarse speckled pattern, with anti Sm and anti ribonucleoprotein (RNP) found in ANA profile examination. Anti‐CCP antibody testing was not done due to limited laboratory panel availability, limiting the serologic evaluation for RA. Myositis‐specific autoantibody (MSA) examination showed positive for anti MDA‐5. Chest CT scan results showed reticular opacities and surrounding ground‐glass opacities in both lungs, some with fibrosis, possibly consistent with CTD‐related interstitial lung disease (CTD‐ILD) (Figure 5). Radiological examination of the wrists and hands found juxta‐articular wrist osteopenia (Figure 4b), which is a nonspecific finding and must be interpreted cautiously, though it was suspected to be an early sign of inflammatory arthritis. The patient was then diagnosed with proximal muscle weakness with dysphagia due to MDA‐5 DM, accompanied by lupus‐like mucocutaneous features and CTD‐related inflammatory arthritis, acting as a clinical mimic of multiple conditions rather than true concurrent overlap diseases.

Initial treatment involved the administration of pulse dose methylprednisolone 2 × 500 mg for 3 days, followed by a gradual dose reduction, hydroxychloroquine 1 × 200 mg, and subsequently mycophenolate sodium 3 × 360 mg after the secondary skin infection resolved. The patient received multidisciplinary care involving neurology, dermatology, gastroenterology, physical medicine and rehabilitation, and clinical nutrition. After treatment, the patient’s motor weakness improved, and he was able to move in bed.

## 3. Discussion

DM is an inflammatory disease with characteristic skin findings and varying degrees of systemic involvement. It is a rare disease with an annual incidence of 1:100,000 people and affects women more frequently. Classic DM (CDM) presents with pathognomonic skin findings and symmetrical, progressive proximal muscle weakness. Skin disease precedes the onset of myositis by 3–6 months in 30%–50% of patients. Classic skin findings may include Gottron’s sign, Gottron’s papules, and heliotrope rash [2].

A thorough physical examination is the basis for making an accurate diagnosis. In this patient, classic DM skin lesions were found, including Gottron’s papules (pinkish‐purple papules on the back of the hands, with a predilection for the skin overlying the metacarpophalangeal and interphalangeal joints) and heliotrope rash (pinkish‐purple erythema involving the upper eyelids, sometimes accompanied by edema). Another characteristic finding is the “V neck sign,” which is a photodistributed or poikilodermatous pinkish‐purple erythema on the anterior neck and upper chest [2]. Other physical findings in this patient included motor weakness consisting of progressive, symmetrical, objective weakness in the proximal lower extremities, neck flexors relatively weaker than neck extensors, and proximal leg muscles relatively weaker than distal muscles, consistent with the criteria in the 2017 ACR/EULAR Classification Criteria for Idiopathic Inflammatory Myopathies [3]. It is clinically important to emphasize that this patient exhibited normal CK levels in the setting of this weakness, a feature commonly seen in MDA‐5‐associated DM phenotypes.

MSAs are exclusively detected in patients with idiopathic inflammatory myopathies, such as DM and polymyositis. MSA‐associated phenotypes can aid in the prognosis and identify potential systemic manifestations. In this case, the patient exhibited anti‐MDA‐5 antibodies. These autoantibodies are more prevalent in Asian populations (11%–57%) compared to Caucasian populations (0%–13%). MDA‐5‐positive DM (MDA‐5‐DM) is a subtype of DM characterized by skin ulceration and an elevated risk of developing ILD, including rapidly progressive variants with high mortality [2, 4]. The presence of ILD in this patient is a typical feature of MDA‐5‐DM, which has the potential to worsen and requires close monitoring, as rapidly progressive ILD (RP‐ILD) dictates a poor prognosis.

One study by Fiorentino et al. [5] described a characteristic skin phenotype in MDA‐5‐DM, including painful erythematous palmar papules and macules, skin and finger ulcerations on top of papules and Gottron’s sign, oral erosions, nonscarring alopecia, and mechanic’s hands. The patient in this case experienced skin ulcerations on the elbows, shoulders, and back, accompanied by alopecia without scarring on the scalp. Skin ulcers in DM often appear in specific areas such as finger joints, elbows, shoulders, knees, ankles, and nail folds and can even appear on the chest, back, arms, hips, throat, or esophagus. These ulcers are usually deep, prominent, and painful. Treatment with conventional glucocorticoids and immunosuppressants is often less effective, so patients require long‐term therapy to achieve remission. In addition, the presence of skin ulceration is a strong indicator of the possibility of ILD in patients with MDA‐5‐DM [4].

Patients with DM have central facial erythema involving the nasolabial folds, unlike the malar erythema in acute cutaneous lupus erythematosus (CLE), which does not affect the nasolabial folds [6, 7]. This patient had multiple erythematous plaques on the forehead, eyebrows, and malar area with nasolabial sparing and discoid lupus features in the form of erythematous lesions with follicular hyperkeratosis and nummular peripheral hyperpigmentation, initially raising suspicion of an overlap disease in the form of SLE. Based on clinical findings in the form of malar rash, discoid rash, laboratory data in the form of low C3, positive ANA 1/320 with a coarse speckled pattern, with borderline anti Sm on ANA profile examination, the patient partially met the SLE criteria (e.g., scoring positive for ANA, low complement, and cutaneous features) using the 2012 SLICC and 2019 ACR/EULAR criteria. However, the diagnosis of SLE in this context appears overstated, as DM can frequently mimic lupus, and the evidence provided is incomplete to confirm two distinct coexisting diseases, pointing instead to a complex mimicry phenotype.

Joint involvement in this patient can be seen more clearly on radiological results in the form of findings of juxta‐articular osteopenia of the right and left wrist. It results from increased bone turnover, driven by inflammatory cytokines. This finding is typical as an early radiographic sign in RA and involves symmetrical bilateral joints [8]. Whereas in DM, nondeforming arthritis with swelling of the distal hand joints can occur. Radiographic features include linear lacy reticular calcification deposits in the skin (40%) with widespread distribution, and osteopenia is not a typical feature of DM [9]. The high RF titer in this patient may be related to RA because the highest frequency of RF is found in RA, approximately 70%–90% [10]. According to 2010 RA ACR/EULAR Classification criteria, the patient had a score of 9/10 (due to joint involvement and elevated RF); however, this diagnosis is weak and insufficiently justified. Anti‐CCP antibody testing was not reported, and there is no evidence of erosive disease or chronic inflammatory arthritis. The clinical diagnosis of RA‐overlap was restricted to seropositive (RF and/or anti‐citrullinated peptide/protein antibody) and erosive patients [11]. Based on the radiologic finding and the classification criteria, this patient is reconsidered to have CTD‐related inflammatory arthritis driven by the primary myositis rather than a definitive RA overlap syndrome.

There are significant potential pitfalls in overclassification. While framing this case as a definitive triple overlap syndrome is tempting, emphasizing how inflammatory arthritis and lupus‐like cutaneous features occur within the broad spectrum of CTD provides greater diagnostic clarity. Distinguishing true overlap syndromes from disease mimicry is important for understanding the disease prognosis and determining appropriate treatment. True overlap syndrome, which by definition meets the criteria for the presence of 2 or more different diseases, is different from mixed CTD (MCTD). MCTD itself is an overlap syndrome that develops and is strongly associated with anti‐U1 RNP antibodies. MCTD combines the overlapping features of SLE, SSc, and PM. This patient did not meet the Sharp, Khan, Kasukawa, or Tanaka criteria [12].

Several studies have shown that idiopathic inflammatory myopathies, which include DM, can overlap with other autoimmune CTDs. CLE with or without systemic manifestations is one of the autoimmune disorders that can overlap with DM [13]. Epidemiologically, the reported autoimmune disease overlap in SLE cases ranges from 17% to 38%. Inflammatory myositis with SLE is reported in 4%–16% of cases. Through laboratory analysis, anti‐RNP myositis‐related antibodies are the most frequently reported in the SLE overlap syndrome with myositis. Lupus‐specific antibodies such as anti‐Sm antibodies are reported more frequently in overlap syndrome than in SLE alone [14].

To fully appreciate the diagnostic complexity of this case, it is crucial to critically evaluate the patient’s clinical and laboratory presentation against established diagnostic guidelines. The profound overlap in symptoms necessitates a careful distinction between true coexisting overlap syndromes and a severe, multisystemic manifestation of a single primary disease. The following assessment outlines how the patient’s features align with the classification criteria for DM, SLE, and RA, highlighting where diagnostic evidence is either well‐supported or overstated, as explained in Table 1.

**Table 1:** Application of classification criteria.

Disease	Criteria supported by findings	Missing evidence/diagnostic concerns
Dermatomyositis (2017 ACR/EULAR) 1	Symmetrical proximal weakness, heliotrope rash, Gottron’s papules, anti‐MDA‐5 positive, myogenic EMG	Normal CK; lacking muscle biopsy or MRI. However, diagnosis is well‐supported clinically
Systemic lupus erythematosus (2019 ACR/EULAR) 2	Positive ANA (1/320), low C3, anti‐Sm/RNP positivity, nonscarring alopecia	Diagnosis is overstated relative to evidence. Mucocutaneous findings heavily overlap with severe MDA‐5‐DM mimicry
Rheumatoid arthritis (2010 ACR/EULAR) 3	Small joint swelling/tenderness, elevated RF	Anti‐CCP testing absent; no erosive disease; juxta‐articular osteopenia is nonspecific. RA diagnosis is insufficiently justified

There are no specific guidelines for the management of MDA‐5‐DM. However, initial therapy for DM usually includes sun protection, antipruritic drugs, and topical anti‐inflammatory drugs. The choice of systemic drugs depends on other DM symptoms, especially myositis or ILD. Glucocorticoids are used as initial therapy, with doses depending on the severity of the disease. The combination of glucocorticoids and other immunosuppressants is commonly used in MDA‐5‐DM with severe skin rash and/or ILD, although there are no studies that determine the best immunosuppressant. The management of DM mimicking overlap syndrome is centered around targeting the primary inflammatory disease, with glucocorticoids as the main therapy. Most patients respond well but require alternative DMARDs to reduce steroid use. In patients who do not respond to steroids, alternative immunosuppressants are needed from the beginning. Methotrexate, azathioprine, cyclosporine, cyclophosphamide, and MMF are usually the second choice in refractory myositis with SLE [4, 14].

Although DM mimicking SLE responds well to corticosteroids, the presence of lung involvement has the potential to cause premature death with an increased standardized mortality ratio compared to SLE alone. Patients with MDA‐5‐DM have the worst prognosis among DM subgroups, especially in the East Asian population where respiratory failure caused by ILD is the main cause of death. In addition, the combination therapy applied can also increase the risk of secondary infection [4, 14].

## 4. Conclusion

Anti‐MDA‐5 DM presenting as a clinical mimic of SLE and RA is a rare and complex condition that requires careful diagnosis and management. A multidisciplinary approach is essential to address the various clinical manifestations and complications associated with the condition. Early diagnosis and aggressive treatment directed at the primary driving pathology can improve the prognosis and quality of life of patients with these multisystem phenotypes.

## Funding

This research received no specific grant from any funding agency in the public, commercial, or not‐for‐profit sectors.

## Consent

Written consent was obtained from the patient.

## Conflicts of Interest

The authors declare no conflicts of interest.

## Data Availability

The data that support the findings of this study are available upon request from the corresponding author. The data are not publicly available due to privacy or ethical restrictions.
